# Frankincense oil-loaded nanoemulsion formulation of paclitaxel and erucin: A synergistic combination for ameliorating drug resistance in breast cancer: *In vitro* and *in vivo* study

**DOI:** 10.3389/fphar.2022.1020602

**Published:** 2022-10-18

**Authors:** Harneetpal Kaur, Kirandeep Kaur, Atamjit Singh, Neena Bedi, Balbir Singh, Mansour S. Alturki, Mohammed F. Aldawsari, Atiah H. Almalki, Shafiul Haque, Hae-Jeung Lee, Dharmendra K. Yadav, Saroj Arora

**Affiliations:** ^1^ Department of Botanical and Environmental Sciences, Guru Nanak Dev University, Amritsar, Punjab, India; ^2^ Department of Pharmaceutical Sciences, Guru Nanak Dev University, Amritsar, Punjab, India; ^3^ Department of Pharmaceutical Chemistry, College of Clinical Pharmacy, Imam Abdulrahman Bin Faisal University, Dammam, Saudi Arabia; ^4^ Department of Pharmaceutics, College of Pharmacy, Prince Sattam Bin Abdulaziz University, Al-kharj, Saudi Arabia; ^5^ Department of Pharmaceutical Chemistry, College of Pharmacy, Taif University, Taif, Saudi Arabia; ^6^ Addiction and Neuroscience Research Unit, College of Pharmacy, Taif University, Taif, Saudi Arabia; ^7^ Research and Scientific Studies Unit, College of Nursing and Allied Health Sciences, Jazan University, Jazan, Saudi Arabia; ^8^ Department of Food and Nutrition, College of Bionano Technology, Gachon University, Seongnam-si, Gyeonggi-do, South Korea; ^9^ College of Pharmacy, Gachon University of Medicine and Science, Incheon City, Korea

**Keywords:** erucin, paclitaxel, synergism, frankincense oil, nanoemulsion, breast cancer

## Abstract

Nanoformulation-based combinational drug delivery systems are well known to overcome drug resistance in cancer management. Among them, nanoemulsions are well-known and thermodynamically stable drug delivery systems suitable for carrying hydrophobic drugs and phytoconstituents to tackle drug-resistant cancers. In the present study, we have investigated the effect of paclitaxel in combination with erucin (natural isothiocyanate isolated from the seeds of *Eruca sativa*) loaded in the frankincense oil-based nanoemulsion formulation. The choice of frankincense oil for the current study was based on reported research investigations stating its magnificient therapeutic potential against breast cancer. Optimized nanoemulsion of paclitaxel (PTX) and erucin (ER) combination (EPNE) provided sustained release and exhibited enhanced cytotoxicity towards human epithelial breast cancer cells (T-47D) as compared to individual ER and PTX. EPNE was further assessed for its antitumor activity in the 7,12-dimethylbenz(a)anthracene (DMBA)-induced breast cancer mice model. EPNE significantly decreased the levels of hepatic and renal parameters along with oxidative stress in breast cancer mice. Furthermore, EPNE also showed decreased levels of inflammatory cytokines TNF-α, IL-6. Histopathological examinations revealed restoration of the tumorous breast to normal tissues in EPNE-treated breast cancer mice. Therefore, EPNE can act as a viable lead and therapeutic option for drug-resistant breast cancer.

## Introduction

**FIGURE 1:**
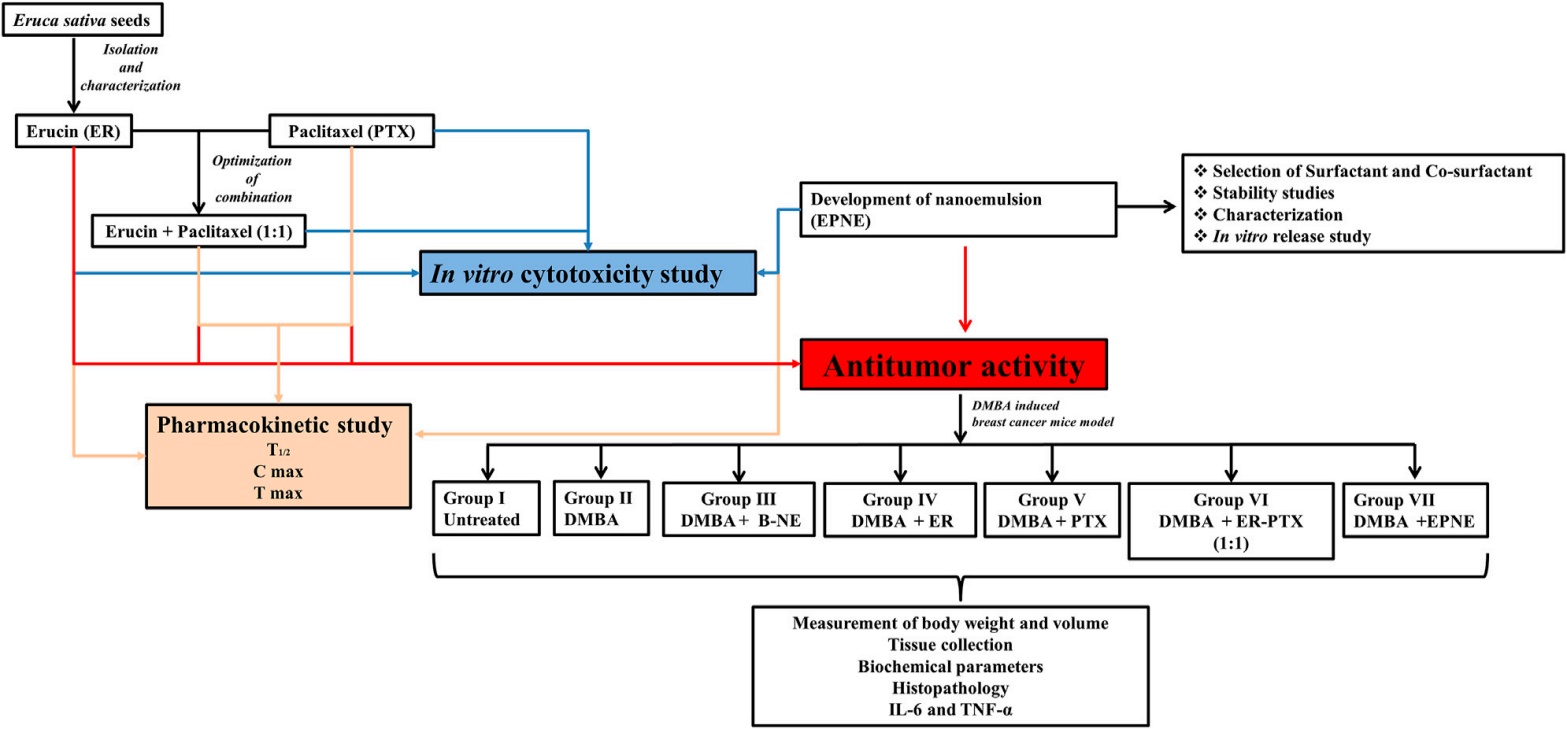
Schematic layout of the study.

Breast cancer is a heterogeneous type of illness that results in the body’s aberrant cells multiplying and dividing out of control, spreading to other body tissues (Testa et al., 2020). The number of new cases of breast cancer has increased from 2.6 lacs (2020) to 3.3 lacs (2022) and the predicted death rate in developed countries rose from 42,280 (2019) to 43,780 (2022) (Bray et al., 2018). Different types of treatment are used for treating breast cancer including surgery, chemotherapy, radiotherapy, and hormone therapy, but these treatment options are becoming ineffective due to the severe side effects and the development of drug resistance (Mitra and Dash, 2018; Noel et al., 2020). The research of novel therapeutic techniques is essential to augment the efficacy of current clinical practices (surgery, chemotherapy, radiotherapy, and hormone therapy). MDR or multidrug resistance development is one of the main reasons for chemotherapy failure (Chen et al., 2015). Thus, there is a dire need to develop an appropriate drug delivery system to improve efficacy and bio-safety in resistant breast cancers. The drug paclitaxel (PTX), is frequently used in clinics to treat a variety of tumor types, including cancers of the breast, pancreas, cervix, ovarian, and other types (Untch et al., 2016; Gawde et al., 2018). However, the prevalence of multiple drug resistance, poor water solubility, and toxic effects restricts the use of paclitaxel as a single agent therapy (Bu et al., 2014). As a result, clinical research is increasingly focused on improving taxane-based regimens by combining them with novel agents. Several reviews have recently been published on nano-sized carriers designed for combination drug delivery in cancer chemotherapy (Eldar-Boock et al., 2013; Luo et al., 2014). Nano-drug delivery is currently being used to tackle drug resistance and toxicity along with convenient administration properties. Nanoemulsions are mixtures comprised of oil and water in which oil droplets are confined to nanometer sizes ranging usually less than 200 nm (Tiwari et al., 2006). Nanoemulsions are widely reported for reducing drug resistance and toxicity of established synthetic drugs in combination with phytoconstituents. Previously prepared nanoemulsion forms of PTX-TPGS (Bu et al., 2014) and PTX-curcumin (Ganta et al., 2010) appeared useful to solve drug resistance problems in breast and ovarian cancer cells, respectively. Moreover, paclitaxel has very low water solubility (less than 1 μg ml−1), and it has been found that paclitaxel was solubilized in alcohol and Cremophor®EL (polyoxyethylated castor oil) for better delivery but suffers from side effects such as hypersensitivity reactions and neuropathy due to the presence of cremophor EL (Wang et al., 2017). Some other PTX formulations are also reported, including Abraxane, Genexol, and Lipusu (Nehate et al., 2014), but they were partially successful to overcome PTX resistance. Thus a novel, effective and safer approach is needed to tackle dose-dependent side effects and multiple drug resistance, keeping this in mind we have prepared nanoemulsion formulation based on a combination of paclitaxel and erucin (a naturally occurring isothiocyanate extracted from the seeds of Eruca sativa) loaded in frankincense oil to address all these limitations. In a recent study, Abd-Rabou and Edris (2022) found that frankincense oil had a strong supportive treatment potential for cancer-related fatigue. Furthermore, other studies also reported that frankincense oil exhibited potent inhibitory activity against different types of cancer cells (Ren et al., 2018; Hakkim et al., 2019). Prepared nanoemulsion formulation (EPNE) was evaluated for its efficacy against DMBA-induced breast cancer in mice (Figure 1).

## Materials and methods

### Chemicals and reagents

Paclitaxel [gift sample from Kwality Pharmaceuticals (Amritsar, India)], Tween 80 was obtained from Merck, India. Transcutol P was acquired from Sigma Aldrich. Frankincense oil was purchased from Devinez (India). All other chemicals and reagents used were of analytical grade. Erucin, used in this study was isolated from the seeds of *Eruca sativa* according to our previously described method (Arora et al., 2016).

### Preparation of nanoemulsion

Phase diagrams were constructed by using the water titration method to recognize the type of structure for emulsification and to typify the behavior of the mixtures with dilution paths. The blank oil-in-water (O/W) NE was prepared under optimal conditions using the water titration method followed by probe sonication. NE was prepared by adding 4% (*v/v*) of Frankincense oil, 36% (*v/v*) of pre-warmed S_mix_ i.e. Tween 20: Transcutol P (3 : 1), and 60% double distilled water. The ratio between oils to surfactants (O/S) was maintained at 1: 9, as this formulation exhibited optimal stability. The resulting mixture was then vortexed for approximately 5 min at room temperature followed by heating and sonication until a clear and transparent solution is obtained (Sonics VCX500 (U.S.A)). The sonication was carried out for approximately 20 min until a clear and transparent phase formed indicating the production of blank nanoemulsion (B-NE). Each experiment was performed in triplicate (*n* = 3). Furthermore, B-NE was incorporated with a combination of active components (ER + PTX) in 1:1 to produce EPNE.

### Physical characterizations of developed nanoemulsion

#### Droplet size analysis

The average droplet sizes (z-average diameter), polydispersity index (PDI), and surface charge (zeta potential) values of B-NE and EPNE samples were identified by dynamic light scattering (DLS) technique through Malvern Particle and Zeta Sizer Analyzer (Zetasizer Nano Series, Worcestershire, United Kingdom). The experiment was performed with a light scattering angle of 173 and at 25°C. All samples were measured in triplicates.

#### Fourier Transform Infrared Spectroscopic

Fourier Transform Infrared Spectroscopic (FTIR) study was done to locate any integral chemical interactions in the test samples ER, PTX, B-NE, and EPNE. The spectra were recorded in Alpha FTIR spectrophotometer (Varian 600-IR Series, Germany) equipped with a deuterated triglycine sulfate detector and a single reflection diamond ATR sampling module. The spectrographs were scanned at the fingerprint region of 400–4,000 cm^−1^ with a 4 cm^−1^ spectral resolution. The data acquisition was performed in *Assistat* 7.7en.

#### Transmission electron microscopy

The reconstituted optimized nanoemulsion (EPNE) was subjected to globule morphology using TEM (JEM-1200 Ex, Joel, Japan). The optimized nanoemulsion (EPNE) (0.1 ml) was reconstituted 100-fold with deionized water. The prepared sample was placed on a carbon-coated grid in a Petri dish and allowed to air dry for about 1 h. The grids were placed in the specimen cabinet and the beam of electrons focused by an electron gun interacted with a small aperture condensed lens. The transmitted samples were focused on charge-coupled device (CCD) camera and images were generated at a magnification of ×15,000 with a fixed X-ray source of 100 cm.

#### Stability studies

The stability constant (KS) was used to assess the stability of optimized nanoemulsion (EPNE). It was determined by the centrifugation-spectrophotometric method as previously described with modifications. The original absorbance of B-NE samples (A_0_) was first measured by using UHPLC (Shimadzu, Kyoto, Japan) at 244 nm and 230 nm for ER, PTX respectively. Then, the centrifugation of 1.0 ml for each sample was performed at 3,000 rpm for 10 min, followed by removing the supernatants. To obtain the sample absorbance (A), the bottom sample was then measured. The Ks value was then calculated as the following equation: Ks = (A_0_–A)/A_0_. It should be noted that a smaller Ks value shows less precipitation of developed nanoemulsion which demonstrates good stability.

#### *In vitro* release studies

The *in vitro* release of developed nanoemulsion and suspensions containing paclitaxel and erucin alone was measured and compared using dialysis bags. Briefly, the samples of paclitaxel (1.25 mg/mL) and erucin (1.25 mg/mL) were loaded into dialysis bags and placed in 50 mL beaker containing the release medium (45 mL of PBS, pH 7.4 containing 0.5% v/v Tween 80). Tween 80 was added to maintain sink conditions. The beakers were then placed for 24 h at 37°C while rotating at 100 rpm using temperature controlled magnetic stirrer. At predetermined time points, 1 mL of each sample was withdrawn and replaced with an equal volume of releasing buffer. The samples were analyzed for paclitaxel and erucin content by HPLC analysis (Arora et al., 2018; Shakhwar et al., 2020).

#### *In Vitro* cytotoxicity study

Human epithelial breast cancer cell line (*T-47D*) and normal mouse fibroblast (*L929*) cells were purchased from the National Centre for Cell Science (NCCS), Pune, India. These cells were cultured in RPMI-1640 and Dulbecco’s Modified Eagle Media (DMEM) respectively with 10% fetal bovine serum (FBS) and maintained at a CO_2_ incubator (5%) at 37°C. Media was exchanged with fresh media at regular intervals. The cytotoxicity of ER, PTX, ER + PTX, B-NE, EPNE was measured against the proliferation of paclitaxel-resistant breast cancer cells (*T-47D*) and normal mouse fibroblast cells (*L929*) (Gomathi et al., 2020). In a 96-well culture plate, cells were seeded (1×10^4^ cells/well) and treated with different concentrations of ER, PTX, ER + PTX, B-NE, EPNE for 24 h. Following treatment, MTT (5 mg/mL) was added to each well and incubated for 4 h in a CO_2_ (5%) incubator. After incubation, each well-received DMSO (100 μL) solubilized formazan crystals that had settled to the bottom. Absorbance was recorded at 570 nm using a multi-well plate reader (BioTek Synergy HT, Winooski, United States). The percentage of growth inhibition was calculated as follows:

$$Inhibition\;of\;Growth\left(\%\right)=\frac{A_{c}-A_{s}}{A_{c}}\times 100$$

Where.

$A_{C}$ , the absorbance of control cells

$A_{S}$ , the absorbance of treated cells

To calculate synergism between erucin with paclitaxel, a combination index was calculated according to Chou and Talalay, 1984. The IC_50_ of combined drugs ER and PTX were taken in 1:1 to calculate the combination index (CI). The CI values for each dose and the corresponding effect level were calculated formula given below:

$$Combination\;index\;\left(CI\right)=\frac{M_{1}}{M_{1x}}+\frac{P_{1}}{P_{1x}}$$

Where.

$M_{1}$ represents conc. of 4-MTBITC (mg/mL) in combination

$P_{1}$ represents conc. of PTX (mg/mL) in combination

$M_{1x}$ represents conc. of 4-MTBITC (mg/mL) alone

$P_{1x}$ represents conc. of PTX (mg/mL) alone

The resulting CI values indicated a quantitative definition for an additive effect (CI = 1), synergism (CI < 1), and antagonism (CI > 1) in drug combinations (Chou and Talalay, 1984). The Fa–CI plot was created by simulating CI values throughout a range of fa levels from 0.1 to 0.95 to provide a visual illustration of drug interactions.Where, fraction affected (fa; the fraction of cells inhibited after exposure to the drug, e.g. 0.5 when cell growth is inhibited by 50%), was calculated.

### Pharmacokinetic studies

Female Balb/c mice were procured from Guru Nanak Dev University, Amritsar, Punjab, India, 143005. The mice were housed in the Central Animal Facility under standard animal husbandry conditions. The experimental protocol was approved by the Animal Ethical Committee of Guru Nanak Dev University (GNDU), Amritsar. The rules and regulations of control and supervision of experimental animals (CPCSEA) were followed according to the Ministry of Environment and Forests (File No. 226/CPCSEA/2022/09), Government of India. Pharmacokinetic studies were carried out using balb/c mice. Animals weighing 25 ± 2 g were fasted for 12 ± 0.5 h with free access to water before initiating the experiment. A dose of 10 mg/kg erucin (dissolved in sunflower oil) as a carrier system, 10 mg/kg of paclitaxel, and developed nanoemulsion were administered by oral gavage. Blood was collected by a retro-orbital route at different time intervals of 0, 0.5, 1, 2, 4, 6, 8, 12, and 24 h. To obtain plasma blood was centrifuged at 15,000 rpm for 10 min. Deproteinization was done by adding acetonitrile to plasma. The supernatant was collected in fresh vials. Erucin and paclitaxel concentrations were analyzed using UHPLC-PDA (Ganta et al., 2010; Arora et al., 2018). In UHPLC analysis low-pressure gradient mode was maintained by using binary mobile phase Acetonitrile: Water (80 : 20) with a flow rate of 0.2 ml/min. The injection volume was 2 μl and the column temperature was set at 60°C. Various pharmacokinetic parameters were analyzed using standard non-compartmental analysis. The area under the plasma concentration-time curve was determined by the linear trapezoidal method. The absorption rate constant (Ka), elimination rate constant (Ke), and absolute bioavailability were calculated using PK Solver Excel Spread Sheet (Version 2.012) (Ganta et al., 2010; Arora et al., 2018).

### *In vivo* experimental studies

Female Balb/c mice (*n* = 42) weighing 24.5 ± 0.5 g were procured from Guru Nanak Dev University, Amritsar, Punjab, India, 143,005. The mice were housed in the Central Animal Facility under standard animal husbandry conditions. The experimental protocol was approved by the Animal Ethical Committee of Guru Nanak Dev University (GNDU), Amritsar. The rules and regulations of control and supervision of experimental animals (CPCSEA) were followed according to the Ministry of Environment and Forests (File No. 226/CPCSEA/2022/09), Government of India. All experiments were performed following ethical standards. After 2 weeks of acclimatization, mice were divided into Groups Ⅰ to VII (*n* = 6). Group I was normal control (does not receive any treatment), Group II was negative control (received DMBA only), Group III was DMBA + placebo (received blank formulation), Group IV received DMBA + erucin (10 mg/kg, p. o.), Group V received DMBA + paclitaxel (10 mg/kg, p.o.), Group VI received DMBA + erucin: paclitaxel (5 mg/kg: 5 mg/kg, p.o.), Group VII received DMBA + nanoemulsion formulation (5 mg/kg: 5 mg/kg, p. o.). DMBA (50 mg/kg, p. o.) was administered weekly for 4–week. After 90 days of DMBA administration (Siddiqui et al., 2013), alternate days of treatment of erucin (Singh et al., 2021; Kaur et al., 2022) and weekly treatment of paclitaxel was given to Group III to VII. Animals were examined daily for morbidity and mortality. The body weight was monitored weekly during the treatment period. Tumor volume was measured and calculated by using the formula: Mean tumor volume = 4/3π*r* ^3^ (where *r* is the mean radius of tumor in mm). At the end of the experiment, the blood sample was withdrawn through retro-orbital plexus puncture and collected for the assessment of biochemical parameters, followed by the sacrifice of all animals through cervical dislocation. The liver and breast tissue were excised from all experimental animals and stored at −20°C for various biochemical and histological analyses (Tartar et al., 2019; Singh et al., 2021).

#### Measurement of biochemical parameters

Blood was centrifuged at 3,000 rpm for 15 min and isolated serum was used to measure the biochemical parameters of hepatic enzymes such as glutamic oxaloacetic transaminase (SGOT), serum glutamic pyruvic transaminase (SGPT), and kidney parameters (urea, creatinine, total bilirubin) and lipid profile (triglycerides, total cholesterol) by using readymade analytical kits (ERBA) (Singh et al., 2021).

#### Estimation of antioxidant status

Homogenate (10% w/v) of liver tissue was prepared in chilled Tris-HCl buffer (pH 8.0) for 5 min, centrifuged at 8,000 rpm for 15 min at 4°C. The supernatant was stored at -80°C for further experimentation. Estimation of oxidative stress parameters in hepatic tissues was done by measuring antioxidant enzymes such as superoxide dismutase (SOD) by following the method of Marklund and Marklund (1974), Catalase (CAT) activity by the method of Sinha (1972) with little modifications. Further, lipid peroxidation in liver tissue homogenate was done by estimating malondialdehyde levels in liver tissue homogenate (Ohkawa 1979).

#### Quantification of pro-inflammatory cytokines

IL-6 and TNF-α were estimated in fresh plasma using respective ELISA kits (Ray Biotech, United States) according to the manufacturer’s instructions.

### Histopathological analysis

Breast tissues were excised from all experimental animals and preserved in 10% formalin solution for various histological analyses. All collected organs were examined for histopathological changes with hematoxylin and eosin (H & E) staining after full fixation in 10% formalin. The tissues were trimmed, embedded in paraffin, sectioned, mounted on microscope slides, and stained with hematoxylin and eosin. Formalin-fixed organs were processed for histopathological examinations (Ramadhani et al., 2021).

### Statistical analysis

The mean standard error is used to express all of the results (SE). The analysis of variances and interactions, with the help of Tukey’s test, was done using a one-way analysis of variance (ANOVA) (*Assistat* 7.7en). The probability *p* ≤ 0.001, 0.01, and 0.05 was carried out to check the statistical significance of all the values at a level of 0.1%, 1%, and 5%.

## Results

### Physical characterization of EPNE formulation

**FIGURE 2:**
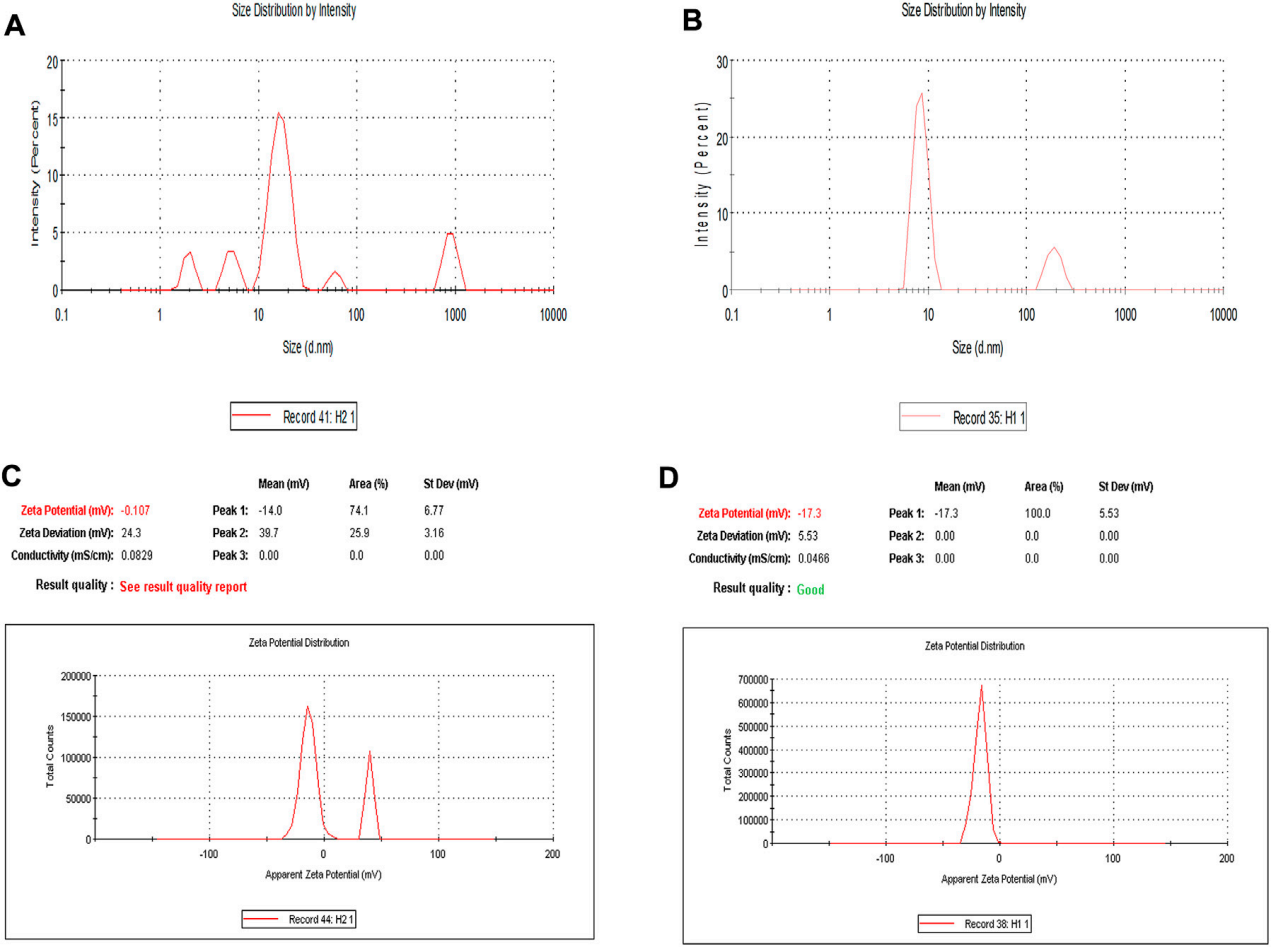
Dynamic light scattering analysis of developed nanoemulsions: Zeta size of **(A)** Blank nanoemulsion (B-NE) **(B)** Drug loaded nanoemulsion (EPNE) **(C)** Zeta potential of blank nanoemulsion (B-NE) **(D)** Zeta potential of drug loaded nanoemulsion (EPNE).

**FIGURE 3:**
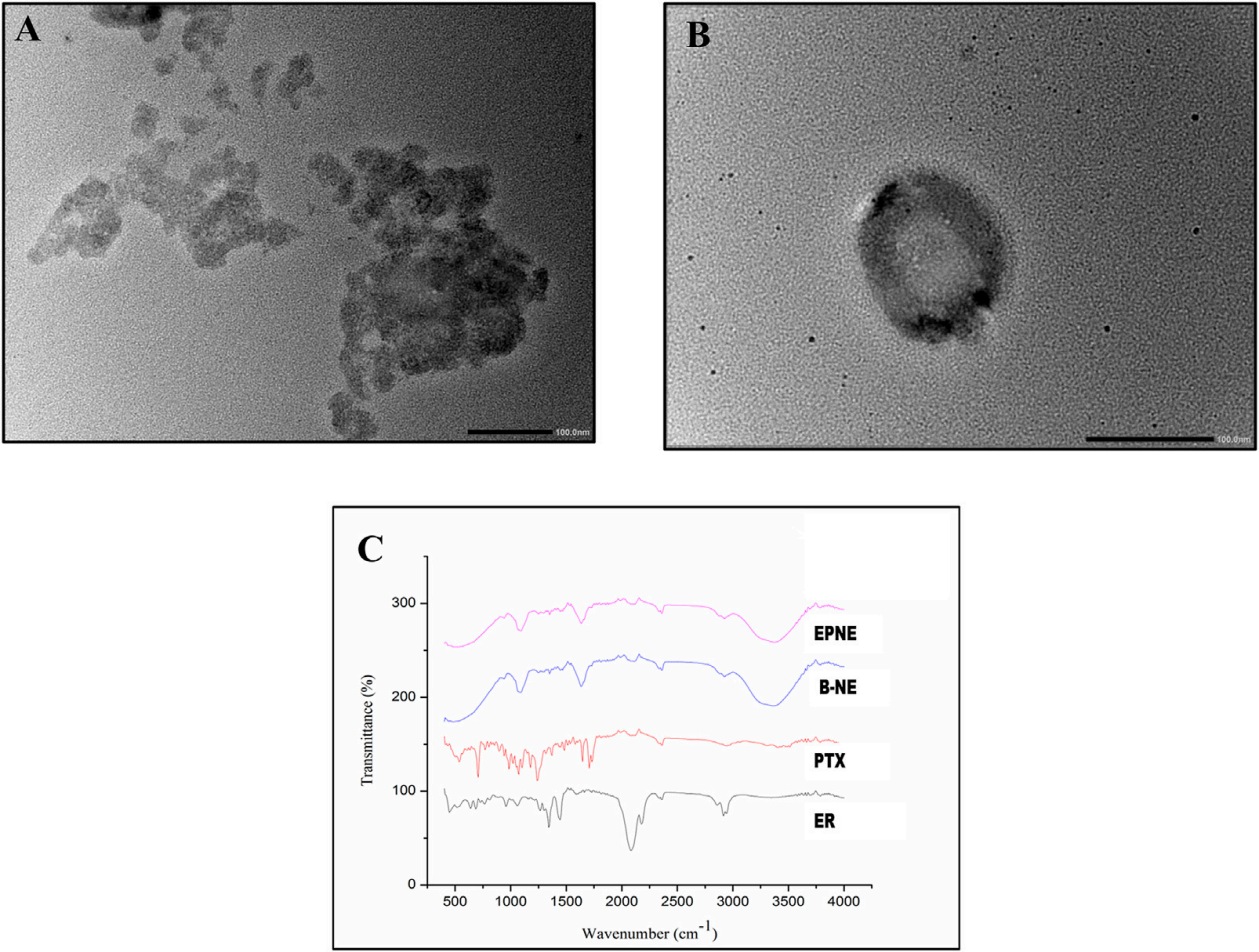
**(A)**, **(B)** showing TEM analysis of developed optimized nanoemulsion (EPNE). **(C)** FTIR analysis of free drugs *viz.* Paclitaxel (PTX), Erucin (ER) and Developed nanoemulsion *viz.* blank nanoemulsion (B-NE), loaded nanoemulsion (EPNE).

To optimize the concentration of surfactant, co-surfactant, and oil in nanoemulsion, pseudo ternary experimentation was performed by using mixtures of frankincense oil and a surfactant mixture of (Tween 20 and Transcutol P) in ratios of 1:1, 2:1, 3:1, 4:1, 5:1, 1:2, and 1:3 and the obtained combination was further used for characterizing various parameters (Supplementary Figure S1). The zeta size, PDI, and zeta potential of developed nanoemulsion possess a crucial part in the functional performance as a delivery system. Supplementary Table S1 depicts that the size of the prepared nanoemulsion was within the desired size range with a narrow size distribution. The zeta size of the developed nanoemulsion loaded with both paclitaxel and erucin was larger than the blank nanoemulsion demonstrating a successful loading of the drug into the system (Figure 2; Supplementary Table S1). However, the zeta potential values of the EPNE were observed to be smaller than the B-NE (Figure 2, Supplementary Table S1). Moreover, the values of stability constant reveal the suitable stability of prepared drug delivery systems (Supplementary Table S2). In FTIR analysis, nanoemulsion did not show any noticeable peak in the spectrum. Although a smooth and widened peak was noticed at 3,369.5 cm^−1^ which denotes the hydrophilic interaction (Figure 3). Furthermore, TEM was performed to examine the physical morphology of the prepared nanoemulsion. The prepared nanoemulsion globules were represented as darker spots with bright backgrounds in carbon-coated grids and were observed to be in a spherical state. In addition, no external particulate of the drug was observed revealing the effective encapsulation of paclitaxel and erucin in the developed system (Figure 3). The obtained outcome of TEM study was reliable with the average globule size of nanoemulsion analyzed by DLS.

### The antiproliferative potential of ER, PTX, ER + PTX, B-NE, and EPNE

**FIGURE 4:**
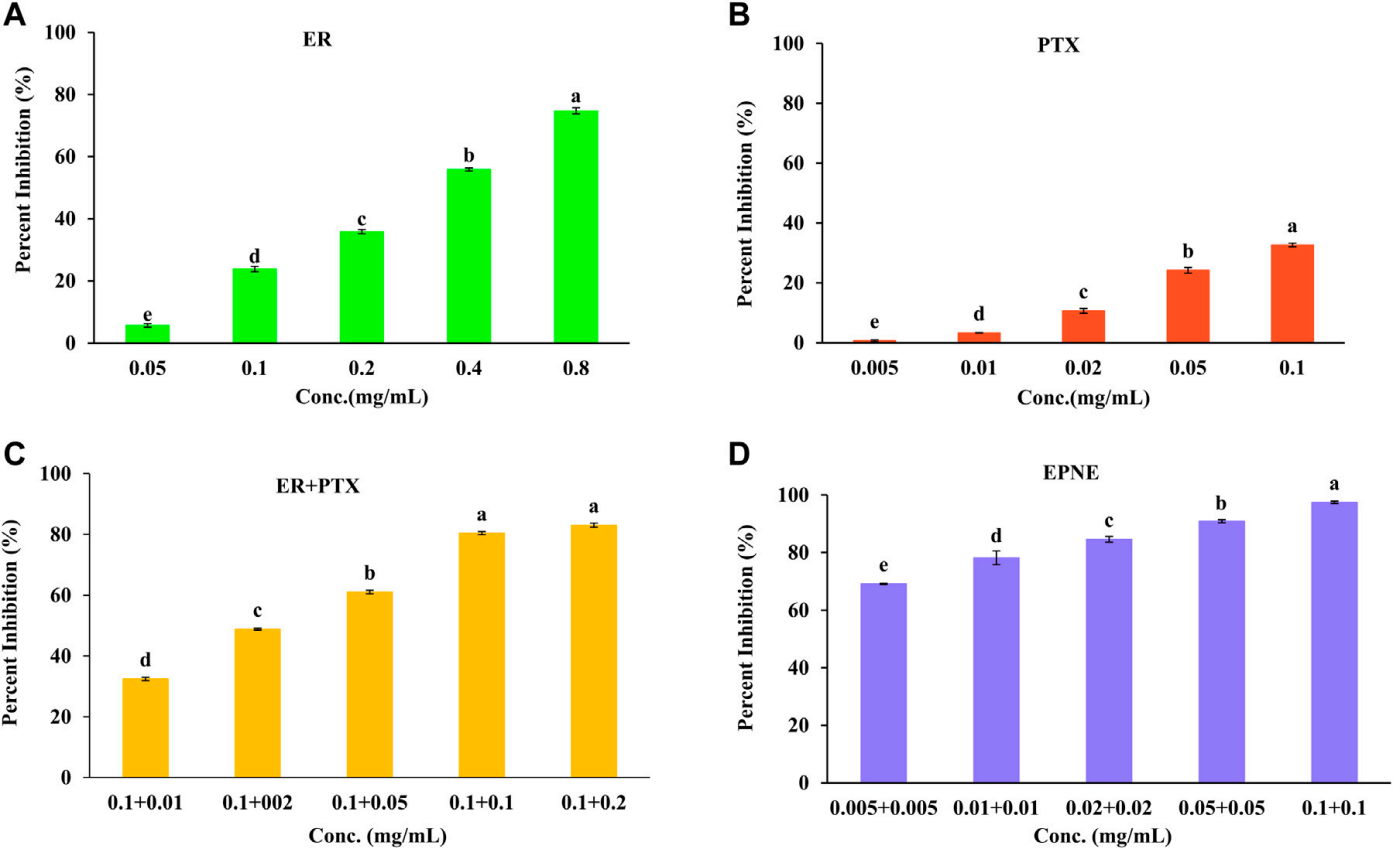
Percent inhibition effect of ER, PTX and their combination on T-47D cells. Cell viability was determined using MTT assay after 24 h treatment. **(A)**Treatment of ER with varying concentrations (0.05, 0.1, 0.2, 0.4, 0.8 mg/ml)) on T-47D cells. **(B)** Treatment of PTX with varying concentrations (0.005, 0.01, 0.02, 0.05, 0.1 mg/ml) on T-47D cells. **(C)** Treatment of mixture of ER + PTX different combinations on T-47 D cells. **(D)** EPNE treatment on cell viability of T-47D cells at 24 h. Data represented as Mean ± SE at the level of significance.

In the present study, an MTT assay was used to check the anti-proliferative potential of ER against human breast cancer cell line T-47D and normal cell line L929. Results demonstrated that ER exhibited the dose-dependent pattern of cytotoxic potential with the minimum IC_50_ values of 0.31 mg/ml against T-47D (Figure 4). Whereas, ER exhibited low toxicity towards normal cell line L929 at its highest concentration of 0.8 mg/ml with an IC_50_ value of 55.68 mg/ml (Supplementary Figure S2).

Moreover, PTX showed cytotoxicity against T-47D cells with IC_50_ value (0.52 mg/ml) as shown in Figure 4. The combination index was calculated and found that better synergistic chemopreventive effects of erucin (ER) and paclitaxel (PTX) in combination were observed on the T-47D cell line. Results showed that by lowering the IC_50_ of ER to ½ times decreases the IC_50_ of synthetic chemotherapy drug paclitaxel (PTX) to ten times (IC_50_ = 0.005 mg/ml). Hence this analysis showed a strong synergistic effect for the combination of ER and PTX (Figure 4; Supplementary Figure S2). Furthermore, B-NE showed 34.3% inhibition of T-47D breast cancer cells. It could may be due to the presence of Frankincense oil (Al-Otaibi et al., 2018; Hakkim et al., 2020). In addition, EPNE demonstrated 97.44 ± 0.52% antiproliferative activity due to the synergistic effect of ER, PTX, Frankincense oil.

### *In-vitro* drug release studies

**FIGURE 5:**
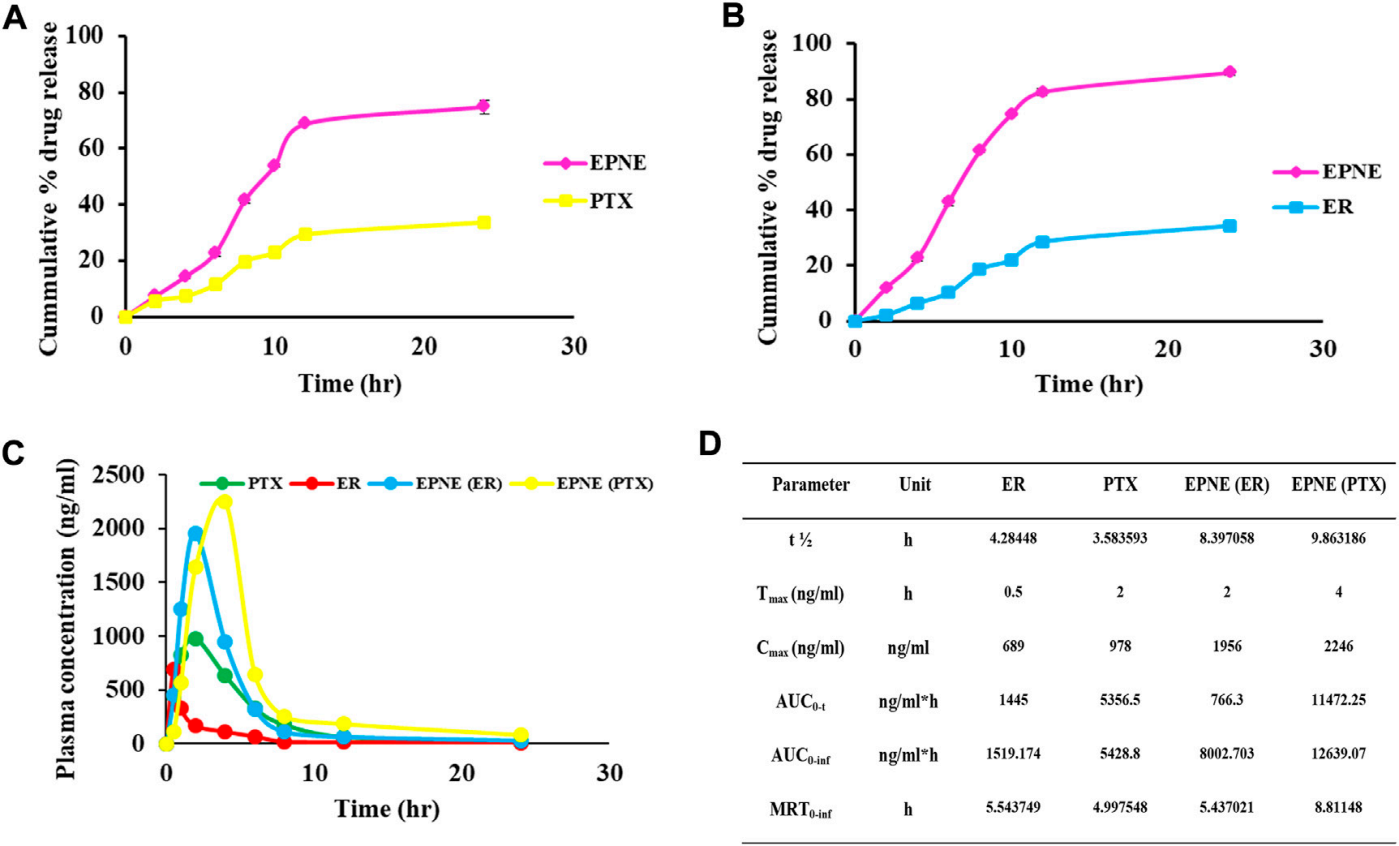
**(A)** Cumulative percentage drug release of paclitaxel (PTX). **(B)** Cumulative percentage drug release of erucin (ER) for optimized nanoemulsion (EPNE) formulation. **(C)** *In vivo* release showed plasma concentration time profile of free erucin, paclitaxel and loaded nanoemulsion **(D)** Pharmacokinetic parameters for erucin, paclitaxel alone and in developed nanoemulsion EPNE in mice.

The release studies revealed initial stage increasing release followed by a constant release pattern of optimized nanoemulsion however drug suspension showed slow release of both the active components i.e. ER and PTX as compared to optimized nanoemulsion formulation (Figure 5).

### Pharmacokinetic studies

From the pharmacokinetic studies, it was found that EPNE significantly enhanced the C_max_ of ER (2.83 folds), and PTX (2.29 folds) while T_max_ was enhanced by four folds of ER and two folds of PTX. t_half_ of both erucin (1.95 folds) and paclitaxel (2.75 folds) was also observed elevated (Figure 5).

### Body weight and tumor volume

**FIGURE 6:**
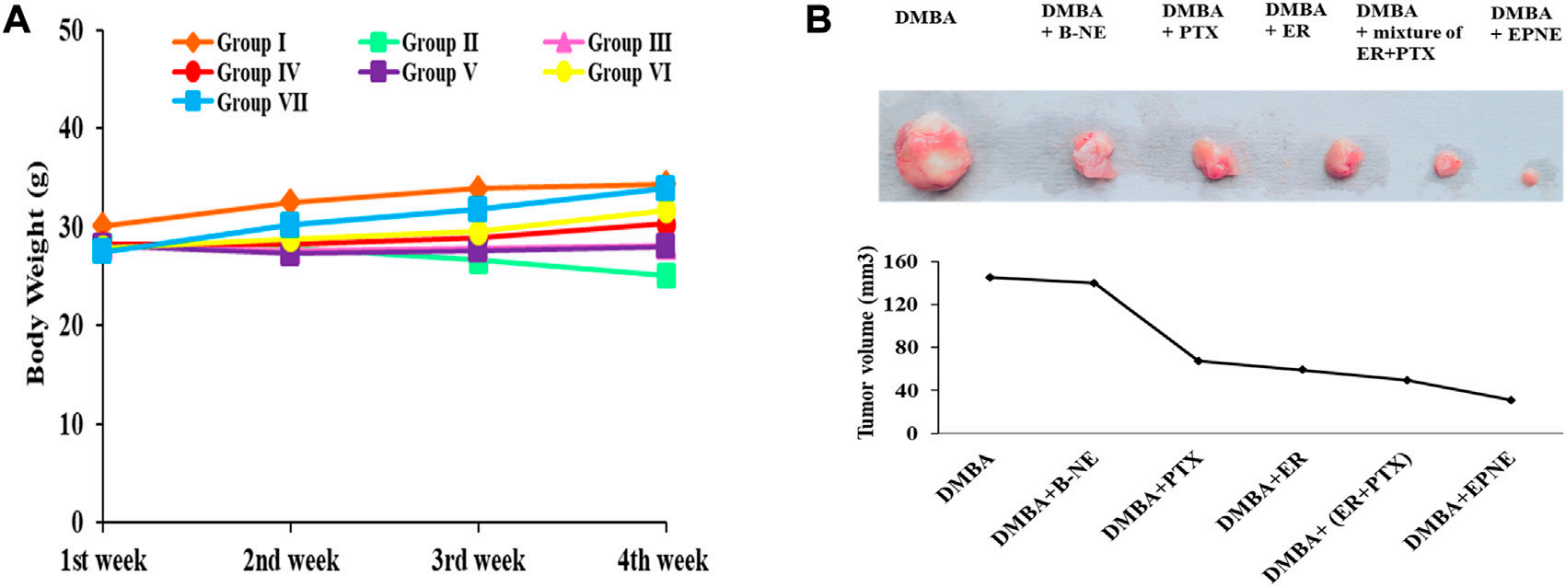
**(A)** Effect of EPNE, free ER, PTX and their 1:1 mixture, Blank nanoemulsion, chemical carcinogen DMBA on the body weight of breast cancer bearing mice during the whole experiment. Group Ⅰ—untreated mice, Group Ⅱ—DMBA treated mice, Group Ⅲ - DMBA + B-NE treated mice, Group Ⅳ—DMBA + ER treated mice, Group Ⅴ—DMBA + PTX treated mice, Group Ⅵ—DMBA + mixture of ER + PTX treated mice, Group Ⅶ—DMBA + EPNE treated mice **(B)** Represented picture of tumors isolated from different groups of breast cancer mice and graph showing tumor volume in the different groups.

Figure 6A depicts the effect of EPNE on body weight. DMBA-treated mice that received EPNE treatment showed a gain in body weight in the last week of the experiment while there was a decline in the body weight of DMBA treated group. Measurement of tumor volume also showed great variation in the EPNE treatment group (30.8 mm^3^) as compared to the free mixture of ER + PTX (49.4 mm^3^), ER (58.9 mm^3^), PTX (67.1 mm^3^) while DMBA treated group showed a tumor of (145.1 mm^3^) as shown in Figure 6B; Supplementary Table S3.

### Estimation of biochemical parameters

Serum parameters were estimated for all the mice. It was found that the hepatic parameters *viz.* SGOT (81.88%), SGPT (85.69%), total bilirubin (169.83%), renal parameters such as urea (116.14%), creatinine (135.74%), and other parameters such as triglyceride (94.15%), cholesterol (103.55%) were significantly enhanced in mice treated with DMBA (group Ⅱ) compared to other groups of ER, PTX, a mixture of ER + PTX and B-NE (Supplementary Table S4). In group Ⅶ, the levels of SGOT were brought down by 41.2%, SGPT by 41.36%, total bilirubin by 61.77%, urea (51.06%), creatinine by 49.06%, triglyceride by 48.31% and cholesterol by 49.12% compared to group Ⅱ (Supplementary Table S4).

### Oxidative stress parameters

**FIGURE 7:**
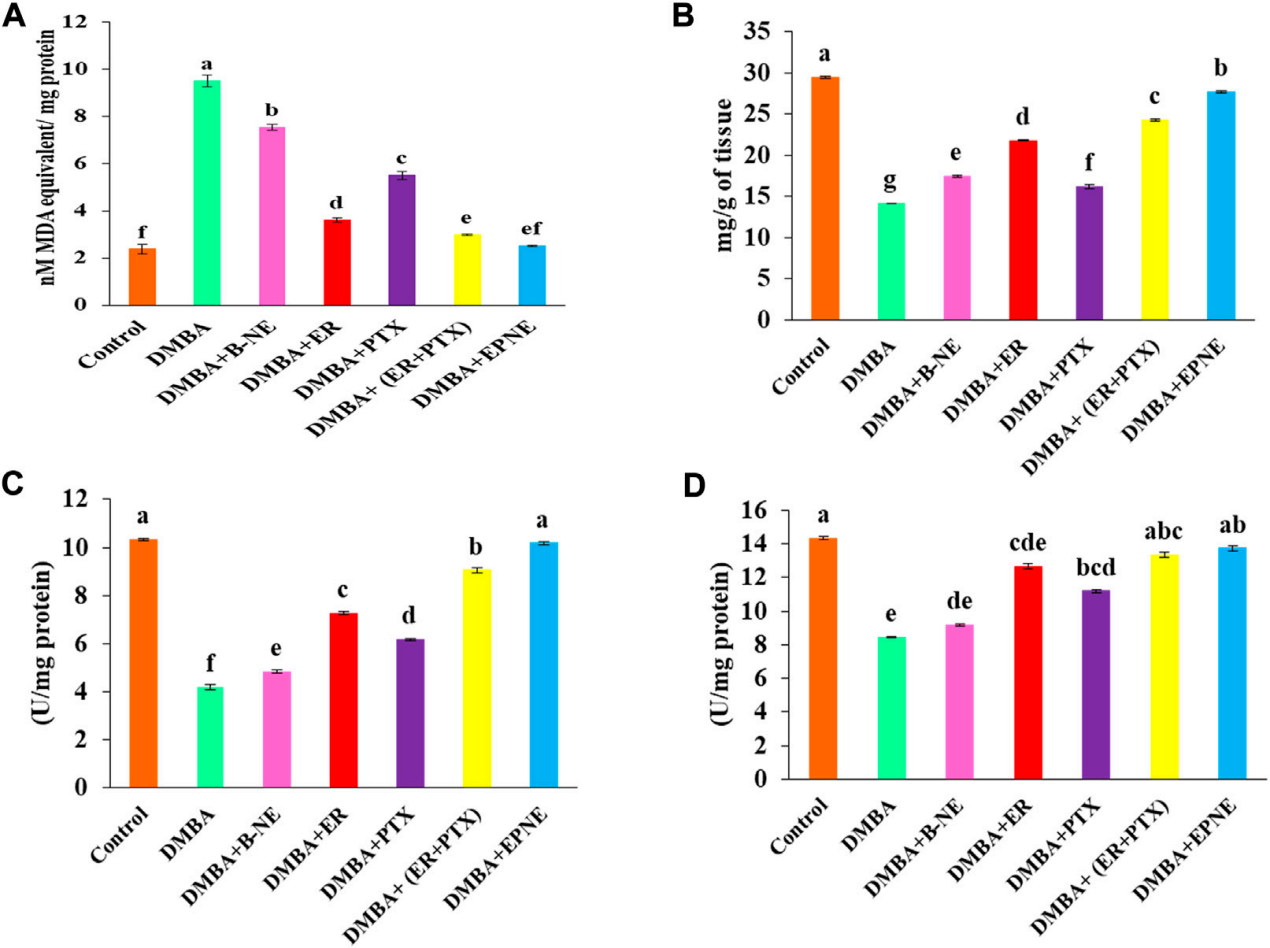
Effect of DMBA on the control group and various experimental group *viz.* ER, PTX, free mixture of ER + PTX, blank nanoemulsion (B-NE) loaded nanoemulsion (EPNE) on oxidative stress parameters. **(A)** The level of lipid peroxidation which indicated nmoles of malondialdehyde per mg protein **(B)** total protein content in the liver homogenate on different experimental groups **(C)** Catalase enzyme activity **(D)** Superoxide dismutase activity. Data represented as Mean ± SE at the level of significance.

DMBA induced toxicity in the liver tissues of tumor-bearing mice by increasing the level of malondialdehyde (9.52 nmole) while EPNE significantly inhibited lipid peroxidation resulting in the decrease in the level of malondialdehyde (2.52 nmole) as compared to the DMBA groups treated with the combination of ER + PTX (2.99 nmole), ER (3.62 nmole), PTX (5.51 nmole), B-NE (7.55 nmole). On the other hand, DMBA generated oxidative stress in the hepatic tissues which in turn results in the decrease of antioxidant enzymes such as SOD (8.44 U/mg of protein), CAT (4.19 U/mg of protein). However, in group Ⅶ, EPNE significantly enhanced the level of antioxidant enzymes by SOD (13.74 U/mg protein), CAT (10.19 U/mg protein) as shown in Figure 7. While other treatment groups of ER, PTX, ER + PTX, B-NE showed the level of SOD, CAT enzymes as (12.66 U/mg protein, 11.19 U/mg protein, 13.35 U/mg protein, 9.2 U/mg protein) and ((7.26 U/mg protein, 6.17 U/mg protein, 9.05 U/mg protein, 4.83 U/mg protein) resp. The total protein content of DMBA, DMBA + B-NE DMBA + ER, DMBA + PTX, DMBA + combination of ER + PTX, DMBA + EPNE is given as 14.14, 17.43, 21.78, 16.16, 24.27, 27.7 mg/g of tissue respectively. Hence the enhanced evel of total protein the EPNE treatment group showed a significant improvement in hepatic cells.

### Pro-inflammatory cytokines

**FIGURE 8:**
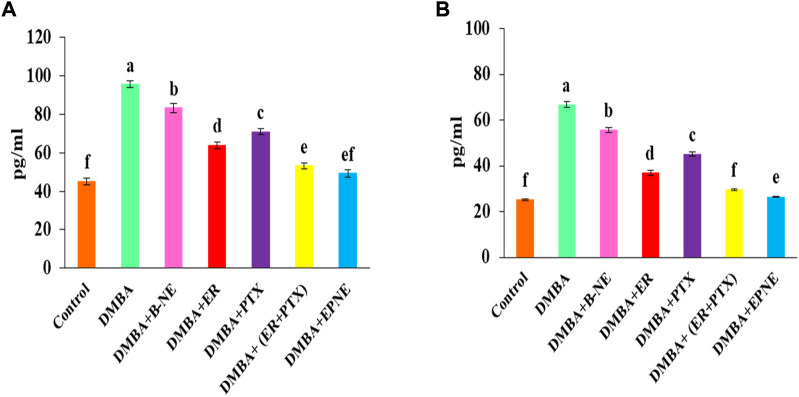
Change in the level of proinflammatory cytokines **(A)** IL-6 and **(B)** TNF-α after the treatment of B-NE, ER, and PTX, free mixture of ER + PTX, EPNE in DMBA-induced breast cancer in mice.

Inflammatory cytokines were also elevated by exposure to DMBA. IL-6, TNF- α are inflammatory markers and their elevated level promotes cancer progression. In our study, DMBA enhanced the level of IL-6 (111.8%), TNF- α (164.76%) in group Ⅱ as compared to the control group (Figure 8). EPNE significantly decreased the level of IL-6 (48.43%), TNF- α (60.32%) as compared to free combination of ER + PTX (44.42%, 55.78%), ER (33.27%, 44.57%), PTX (25.78%, 32.34%) and B-NE (12.89%, 16.47%) resp. in the breast cancer-bearing mice.

### Histopathology

**FIGURE 9:**
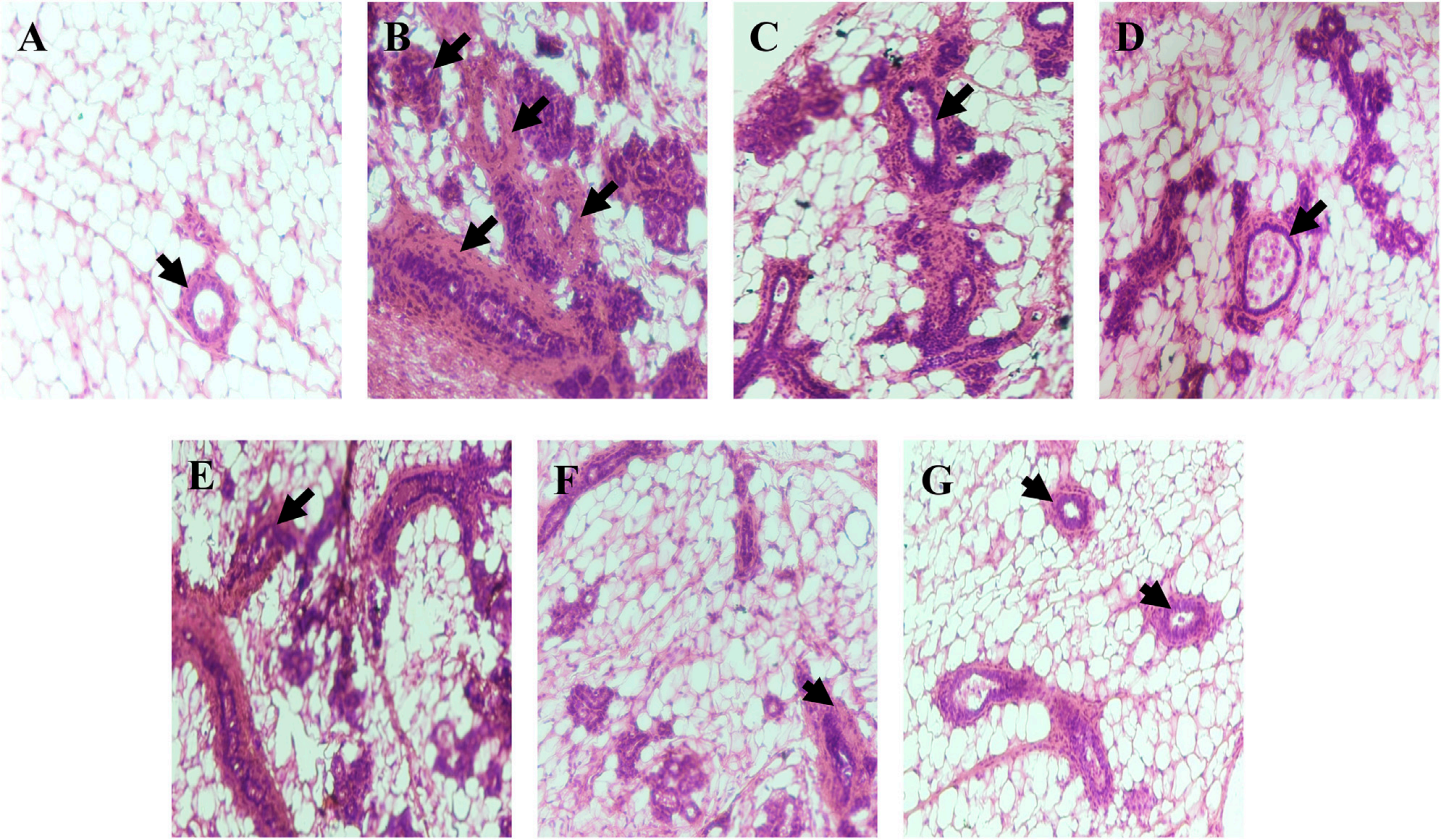
Histopathological examination: **(A)**. Mammary tissue of control group showing normal acini and ductules (black arrow); **(B)**. Mammary tissue of DMBA administered group showing excessive proliferation of epithelial lining of acini and ductules with hyperchromatic nuceli (black arrow); **(C)**. Mammary tissue of DMBA + B-NE administered group; **(D)**. Mammary tissue of DMBA + ER administered group; **(E)**. Mammary tissue of DMBA + PTX administered group; **(F)**. Mammary tissue of DMBA + combination of ER + PTX administered group; **(G)**. Mammary tissue of DMBA + EPNE administered group showing restoration to normal tissue with normal acini (black arrow).

In histopathological analysis, mammary tissue of the control group showed normal acini and ductules. However DMBA administered group showed excessive proliferation of epithelial lining of acini and ductules with hyperchromatic nuclei (black arrow); mammary tissue of DMBA + Placebo nanoemulsion administered group showed slight improvement while mammary tissue of DMBA + erucin administered group, mammary tissue of DMBA + paclitaxel administered group and DMBA + (ER + PTX) mixture administered group showed little more improvement in the proliferation of epithelial lining of acini and ductules with less hyperchromatic nuclei as compare to only DMBA treated group. Furthermore, DMBA + nanoemulsion administered group showed restoration to normal tissue with normal acini (black arrow) (Figure 9).

## Discussion

In the present study paclitaxel (PTX), erucin (ER), and frankincense oil were the therapeutically efficient components decorating together the optimized nanoemulsion. The developed nanoemulsion (EPNE) was evaluated for its antitumor efficacy. The prepared nanoemulsion showed zeta size of B-NE was 29 ± 0.71 nm, EPNE was 18.1 ± 0.5 nm and zeta potential of B-NE was −0.107 mV, EPNE was −17.3 mV. TEM analysis of EPNE revealed the spherical and nanosize emulsion globules efficiently encapsulating paclitaxel and erucin within a layer of frankincense oil further surrounded by the continuous aqueous phase. *In vitro* release profile further demonstrated a sustained release pattern of paclitaxel and erucin from nanoemulsion formulation when compared with erucin and paclitaxel suspension alone. EPNE showed excellent growth inhibition (97.44 ± 0.52%) of paclitaxel-resistant estrogen-positive human breast cancer cells T-47D and showed minimum cytotoxicity in normal cell line L929 as compared to T-47D cells.

*In vivo,* pharmacokinetic studies showed enhanced plasma concentrations of both erucin as well as paclitaxel. Potent results of *in vitro* cytotoxicity studies and *in vivo* pharmacokinetic outcomes established the solid ground for further assessment of EPNE for its *in vivo* antitumor potential against breast cancer. DMBA (7,12-Dimethylbenz(α)anthracene) induced breast cancer model is an important preclinical animal model as it mimics morphological, histopathological, and biochemical features of human breast cancer (Liu et al., 2015; Arif et al., 2019). Therefore DMBA-induced breast cancer in the Balb/c mice model was chosen for further evaluation of the anti-tumor activity of EPNE. After 120 days, EPNE treatment showed a reduction in tumor size (30.8 mm^3^) as compared to free PTX (67.1 mm^3^), ER (58.9 mm^3^), and the mixture of ER + PTX (49.4 mm^3^) while the DMBA treatment group showed tumor volume of 145.1 mm^3^. During treatment, significant improvements in body weight were observed in the EPNE-treated groups as compared to groups of DMBA, ER, and PTX solution. Furthermore, histopathological analysis confirmed the restoration of breast tumor in the EPNE-treated group to normal tissues at the end of the experiment. The findings of the current study also found that DMBA elevated the levels of liver serum transaminases i.e., SGOT, and SGPT in the mice serum, resulting in hepatic injury. From the previous study, it was found that DMBA-induced liver damage triggers the release of SGOT, SGPT, in the bloodstream which indicated hepatocellular damage. Similarly in the present study, the level of SGOT, and SGPT were significantly increased in group Ⅱ by 81.88%, 85.69% resp. However, EPNE treatment altered this effect by decreasing the level of SGOT, and SGPT by 41.2%, and 41.36% respectively in the serum. DMBA toxicity induces damage in the kidney tissues resulting in elevated urea, creatinine, total bilirubin, cholesterol, and triglycerides level in the bloodstream, but EPNE treatment significantly decreases the level of kidney parameters by 51.06%, 49.06%, 61.77%, 49.12%, and 48.31% respectively in the serum which in turn showed the renal protective effect. However, the promising renal-protective effect is related to the efficacy of EPNE. The process of DMBA carcinogenesis comprises alteration in the tissue redox balance, resulting in oxidative stress which is responsible for biochemical and pathophysiological disturbances in rats (Lai and Singh 2006; Krishnamoorthy & Sankaran 2016). In the present study, DMBA administration increased oxidative stress in the hepatic cells by increasing the level of MDA and declining the levels of antioxidant enzymes such as SOD, and CAT in the liver homogenate. Superoxide dismutase and catalase enzymes catalyze cellular defense systems against oxidative damage caused by free radicals. Group Ⅶ showed a significant decrease in the level of MDA and an increase in the level of SOD, and CAT, which indicated the protective effect of the optimized nanoemulsion. The increase in total protein content in the EPNE-treated group shows that EPNE was effective in improving liver cells. Since EPNE improves the functional status of the liver cells and increases protein production in damaged liver tissue. Inflammation also plays a very important role in the progression of cancer (Grivennikov et al., 2010). Interleukin-6 (IL-6) and tumor necrosis factor (TNF), two pro-inflammatory indicators, rise in response to infection, tissue injury, and states of active stress like obesity (Ilyasova et al., 2005). The overexpression of these markers can cause genomic instability, uncontrollable cellular division, and cellular membrane damage. They may also boost estrogen levels in breast tissue and block the antitumor immune response, all of which may work together to directly enhance the stages of neoplastic transformation through increasing breast density (Hanna et al., 2017). Gyamfi et al., 2018 and Sheng et al., 2018 reported that the pro-inflammatory cytokine IL-6 has been directly linked to breast cancer progression and risk. DMBA toxicity is known to trigger the release of inflammatory cytokines such as TNF-α, and IL-6 in breast cancer (Ramadhani et al., 2020). EPNE significantly downregulated the level of inflammatory markers such as TNF-α, and IL-6 as compared to groups of the combination of ER + PTX, ER, and PTX alone in DMBA induced breast cancer group.

## Conclusion

Paclitaxel and erucin-based nanoemulsion formulation was prepared for targeting breast cancer that exhibited potent cytotoxic activity against paclitaxel-resistant T-47D breast cancer cells. Optimized nanoemulsion (EPNE) exhibited an improved pharmacokinetic profile resulting in enhanced bioavailability of both paclitaxel and erucin. Enhanced C_max_ and T_max_ of EPNE will certainly reduce the potency, dosing frequency, and patient compliance. *In vivo* assessment proved the capability of EPNE to restore the cancerous breast tissue to normal tissue. EPNE treatment also showed improved levels of biochemical parameters and promoted an antioxidant defense system. Furthermore, treatment of EPNE also showed a reduction in the levels of inflammatory cytokines. The overall outcome suggested the capability of EPNE to become a viable lead for combinatorial drug delivery and future therapeutic option for drug-resistant breast cancer.

## Data Availability

The original contributions presented in the study are included in the article/Supplementary Material, further inquiries can be directed to the corresponding authors.
