# Supplementary material for: Frankincense oil-loaded nanoemulsion formulation of paclitaxel and erucin: A synergistic combination for ameliorating drug resistance in breast cancer: In vitro and in vivo study
Source: Front Pharmacol. 2022 Oct 18;13:1020602. doi: 10.3389/fphar.2022.1020602 (PMC9623270; doi:10.3389/fphar.2022.1020602)
Supplement: Supplementary file 1 [file DataSheet1.docx]

Supplementary Material

**Supplementary Table 1. Physical characteristics of the EPNE formulation.**

| **Formulation**  **code** | **Z-average diameter (nm)** | **Polydispersity index (PDI)** | **Zeta potential (mV)** |
| --- | --- | --- | --- |
| **B-NE** | **29±0.71** | **0.035±0.010** | **-0.107** |
| **EPNE** | **18.1±0.5** | **0.054±0.021** | **-17.3** |

The data were expressed as the mean ± SE. Significance level ****p* ≤ 0.001, ***p* ≤ 0.01. the blank B-NE vs. the corresponded EPNE.

**Supplementary Table 2. Stability constant of optimized Nanoemulsion (EPNE)**

| **Serial No.** | **Component** | **Stability constant (Ks)%** |
| --- | --- | --- |
| 1. | Frankincense | 0.090± 0.006 |
| 2. | Paclitaxel | 0.023± 0.003 |
| 3. | Erucin | 0.369± 0.05 |

The data were expressed as the mean ± SE. Significance level ****p* ≤ 0.001, ***p* ≤ 0.01.

**Supplementary Table 3. Effect of EPNE on the tumor size of DMBA induced breast cancer mice**

| **Groups** | **Treatment** | **Mean Tumor Volume (mm^3^) ±S.E.** |
| --- | --- | --- |
| **Ⅱ** | **DMBA** | 145.13 ± 1.35 |
| **Ⅲ** | **DMBA+B-NE** | 139.63± 0.37 |
| **Ⅳ** | **DMBA + PACLITAXEL** | 67.16 ± 0.76 |
| **V** | **DMBA + ERUCIN** | 58.93 ± 0.33 |
| **VI** | **DMBA + Mixture of ERUCIN + PACLITAXEL** | 49.4 ± 0.22 |
| **VII** | **DMBA + NANOEMULSION** | 30.83 ±0.48 |

The data were expressed as the mean ± SE. Significance level ****p* ≤ 0.001, ***p* ≤ 0.01.

**Supplementary Table 4. Effect of EPNE on the different biochemical parameters**

| **Groups** | **Treatment** | **SGOT (U/L) ± S.E.** | **SGPT (U/L) ± S.E.** | **Total Protein**  **(U/ml)** | **Total**  **Bilirubin**  **(mg/dl) ± S.E.** | **Urea (mg/dl)**  **± S.E.** | **Creatinine (mg/dl) ± S.E.** | **Triglycerides (mg/dl) ± S.E.** | **Total cholesterol (mg/dl) ± S.E.** |
| --- | --- | --- | --- | --- | --- | --- | --- | --- | --- |
| Ⅰ | **Control** | 154.49 ± 1.49 | 64.3 ±  1.26 | 4.82 ±  0.03 | 0.40 ±  0.008 | 20.92 ±  0.39 | 0.415 ±  0.009 | 34.81 ±  0.71 | 141.43 ±  0.30 |
| **Ⅱ** | **DMBA** | 281.66 ±  6.30 | 119.4 ± 1.91 | 1.92 ±  0.02 | 1.08 ±  0.03 | 45.23 ±  0.33 | 1.278 ±  0.105 | 67.59 ±  0.74 | 287.90 ±  0.61 |
| **Ⅲ** | **DMBA+B-NE** | 255.53 ±  2.68 | 101.44 ±  1.06 | 2.17 ±  0.02 | 0.92 ±  0.01 | 35.89 ±  0.96 | 0.91 ±  0.01 | 55.66 ±  0.69 | 246.19 ±  1.27 |
| **IV** | **DMBA + PACLITAXEL** | 206.58 ± 1.17 | 62.48 ±  0.41 | 3.83 ±  0.07 | 0.54 ±  0.01 | 30.37 ±  0.37 | 0.735 ±  0.015 | 42.085 ±  0.54 | 187.16 ±  1.14 |
| **V** | **DMBA + ERUCIN** | 244.60 ± 2.64 | 85.25 ±  1.28 | 2.80 ±  0.04 | 0.65 ±  0.01 | 32.58 ±  0.24 | 0.83 ±  0.007 | 48.29 ±  0.39 | 204.93 ±  0.37 |
| **VI** | **DMBA + Mixture of ERUCIN + PACLITAXEL** | 182.29 ± 3.38 | 72.54 ±  0.88 | 4.16 ±  0.04 | 0.46 ±  0.01 | 25.78 ±  0.38 | 0.601 ±  0.01 | 39.09 ±  0.34 | 162.87 ±  0.38 |
| **VII** | **DMBA + NANOEMULSION** | 165.53 ± 1.20 | 70.005 ±  0.64 | 4.62 ±  0.02 | 0.41 ±  0.006 | 22.13 ±  0.31 | 0.498 ±  0.007 | 34.93 ±  0.29 | 146. 46 ±  0.18 |

The data were expressed as the mean ± SE. Significance level ****p* ≤ 0.001, ***p* ≤ 0.01.

**
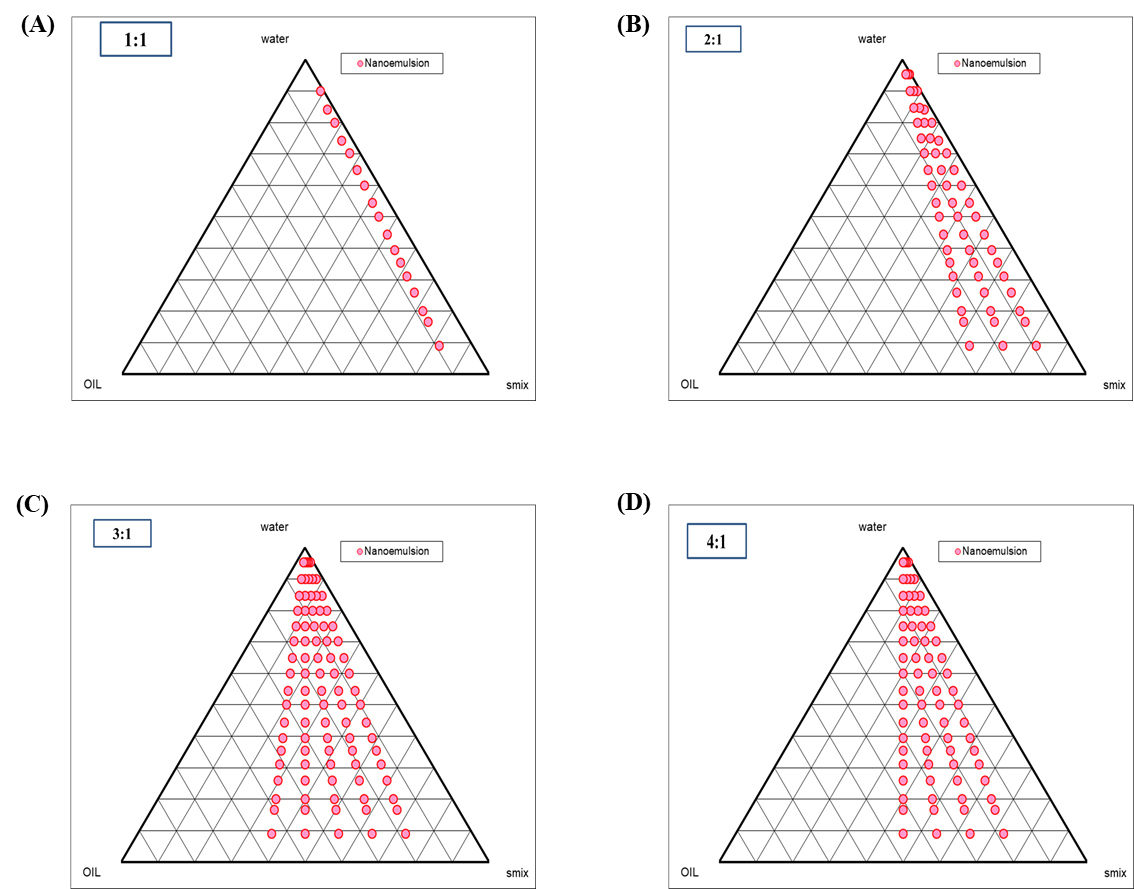
**

**Supplemenatry Figure S1:** Pseudoternary phase diagrams constucted using Frankinsence oil and Tween 20 and Transcutol P as surfactant and co-surfactant in ratios of 1:1, 2:1, 3:1 and 4:1 when titrated further for 5:1 and 6:1 ratios it showed gelation moreover all the nanoemulsion titrated in surfactant mixture in 1:2 and 1:3 ratios were found turbid. Surfactant ratio of 3:1 showed wider nanoemulsion region hence selected further for developing drug loaded nanoemulsion.


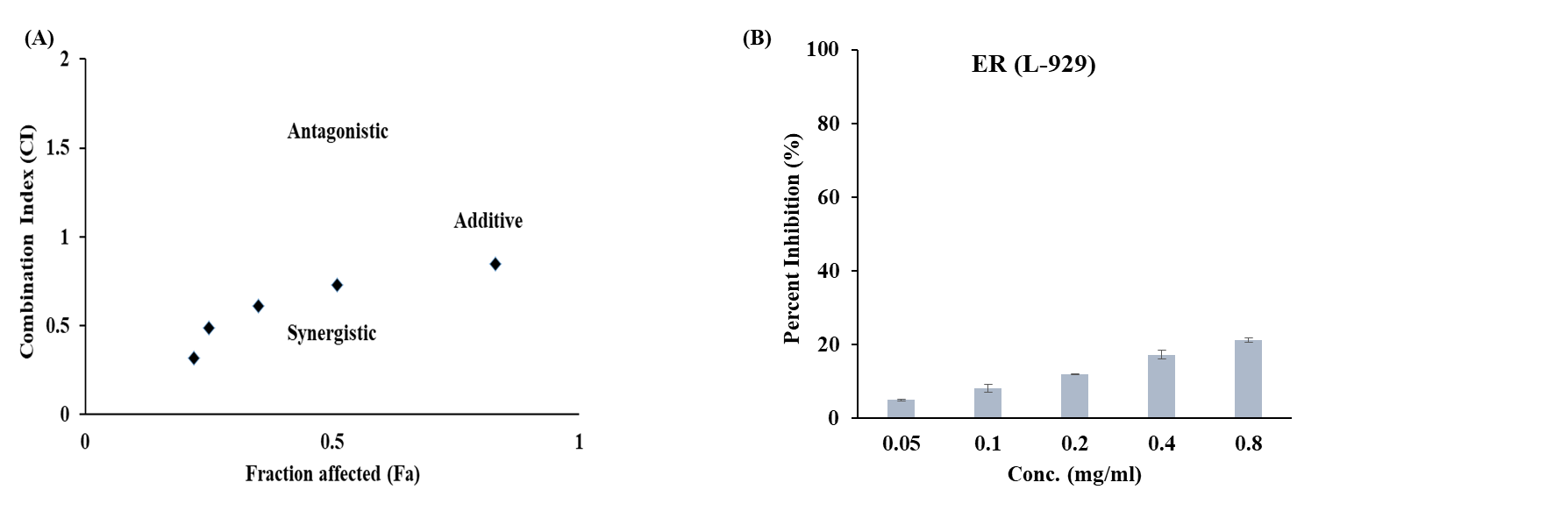


**Supplementary Figure S2:** (A) Combination Index Plot. CI > 1, CI = 1, and CI < 1 indicate antagonistic, additive, and synergistic effects. (B) Effect of erucin on normal human fibroblast cells (L-929).
